# Tool, weapon, or white elephant? A realist analysis of the five phases of a twenty-year programme of occupational health information system implementation in the health sector

**DOI:** 10.1186/1472-6947-12-84

**Published:** 2012-08-06

**Authors:** Jerry M Spiegel, Karen Lockhart, Carmen Dyck, Andrea Wilson, Lyndsay O’Hara, Annalee Yassi

**Affiliations:** 1 Global Health Research Program (GHRP), School of Population and Public Health, University of British Columbia (UBC), Vancouver BC V6T 1Z3, Canada

## Abstract

**Background:**

Although information systems (IS) have been extensively applied in the health sector worldwide, few initiatives have addressed the health and safety of health workers, a group acknowledged to be at high risk of injury and illness, as well as in great shortage globally, particularly in low and middle-income countries.

**Methods:**

Adapting a context-mechanism-outcome case study design, we analyze our team’s own experience over two decades to address this gap: in two different Canadian provinces; and two distinct South African settings. Applying a realist analysis within an adapted structuration theory framing sensitive to power relations, we explore contextual (socio-political and technological) characteristics and mechanisms affecting outcomes at micro, meso and macro levels.

**Results:**

Technological limitations hindered IS usefulness in the initial Canadian locale, while staffing inadequacies amid pronounced power imbalances affecting governance restricted IS usefulness in the subsequent Canadian application. Implementation in South Africa highlighted the special care needed to address power dynamics regarding both worker-employer relations (relevant to all occupational health settings) and North–south imbalances (common to all international interactions). Researchers, managers and front-line workers all view IS implementation differently; relationships amongst the workplace parties and between community and academic partners have been pivotal in determining outcome in all circumstances. Capacity building and applying creative commons and open source solutions are showing promise, as is international collaboration.

**Conclusions:**

There is worldwide consensus on the need for IS use to protect the health workforce. However, IS implementation is a resource-intensive undertaking; regardless of how carefully designed the software, contextual factors and the mechanisms adopted to address these are critical to mitigate threats and achieve outcomes of interest to all parties. Issues specific to IS development, including technological support and software licensing models, can also affect outcome and sustainability – especially in the North–south context. Careful attention must be given to power relations between the various stakeholders at macro, meso and micro levels when implementing IS. North–South-South collaborations should be encouraged. Governance as well as technological issues are crucial determinants of IS application, and ultimately whether the system is seen as a tool, weapon, or white elephant by the various involved parties.

"You may call me a fool, But was there a rule The weapon should be turned into a tool? And what do we see? The first tool I step on Turned into a weapon. **- Robert Frost**"

"White (albino) elephants were regarded as holy in ancient times in Thailand and other Asian countries. Keeping a white elephant was a very expensive undertaking, since the owner had to provide the elephant with special food and provide access for people who wanted to worship it. If a Thai King became dissatisfied with a subordinate, he would give him a white elephant. The gift would, in most cases, ruin the recipient. - **The Phrase Finder**"

## Background

Despite expenditure of billions of dollars worldwide in information system (IS) applications, controversy persists concerning what this extensive investment has achieved [1-4]. While IS implementation must ultimately be assessed with regard to applications in specific social contexts and not in the abstract, this orientation has largely been neglected [5,6]. To address this challenge, the *structurationist* theoretical orientation [7,8] emphasizes that each IS configuration decision “is not merely technical, but social and political, affecting end-users’ practices” [8]. However, consideration of micro-, meso- and macro-level influences on relevant outcomes has received limited consideration [9] and the structurationist framing itself has been the subject of considerable critique, including shortcomings in adequately appreciating the nuances of contextual factors [10]. Furthermore, while Myers & Klein observed that IS applications are increasingly addressing “social issues such as freedom, power, social control, and values with respect to the development, use, and impact of information technology” [11], the direct engagement in IS implementation of those whose health is directly in question still remains largely ignored. This contrasts with the active debate that has occurred regarding how geographic information systems have been implemented in circumstances where residents of affected communities have felt victimized when their involvement has been marginalized [12,13].

Among those cognizant of the seminal importance of context, the power implications related to how a “surveillance gaze” is applied have stimulated much reflection [14]. Critical theorists have given explicit consideration to how resistance to intended IS application may be manifest, such as by health professionals who attempt to adapt its application to their own perceived interests [14] or by those who respond to being observed by seeking to disrupt smooth functioning and control [15]. However, in addition to ambiguities rooted in whether an IS is perceived from the perspective of specific groups as either contributing value (as a “tool”) or threatening particular interests (as a “weapon”), IS technology transfer is always fraught with the danger of being a “white elephant” that confers limited benefit and drains resources.

To mitigate obstacles to access restrictions that can be provoked by proprietary ownership of intellectual property (not unlike the debates regarding generic drugs as an option to reliance on patent protection of pharmaceuticals), IS innovations have also triggered development of alternative licensing and knowledge-sharing orientations. These include Creative Commons licensing, in which software products are made available with the requirement of attribution, non-commercialization, and either no derivatives allowed, or a share-alike model, in which modifications and new developments are then shared with “the commons” [16]. This is potentially an important strategy in mitigating power dominance. “Open Source Software” (OSS), which operates on this principle, nevertheless still requires a dedicated international group of skilled developers, or at least a well-resourced passionate host to maintain the system [17]. Moreover, Hertel *et al.* note that even in the OSS community, contributors’ motivations to OSS may primarily be to improve their own software (i.e. learning opportunities) and participation (i.e. as part of a large team), and still not necessarily involve ultimate users of the software [17].

Another important contextual factor, one that reflects North–South power imbalances in particular, is the “digital divide”, which refers to the wide disparities that currently exist between high income countries (HICs) that have developed IS solutions in comparison to low and middle income countries (LMICs) where capacities for developing and applying such undertakings may be quite limited [18,19]. Studies on information technology in LMICs [20-23] repeatedly emphasize the need to better understand how to best introduce health IS for decision-making in these settings – which is of particular relevance given the disproportionately greater global disease burden that occurs here.

Ultimately, as Guba & Lincoln observed, stakeholders’ assessment of an intervention’s worth largely depends on how the causes of underlying problems are perceived [24]. In this regard, those who design a particular intervention, or govern its development and use, typically do this with a different rationale or perspective than those who are affected; and designers, decision-makers, and deliverers might themselves maintain distinct norms and values [25]. Accordingly, the power relations embedded in settings where information systems are used must be addressed [26].

**Figure 1:**
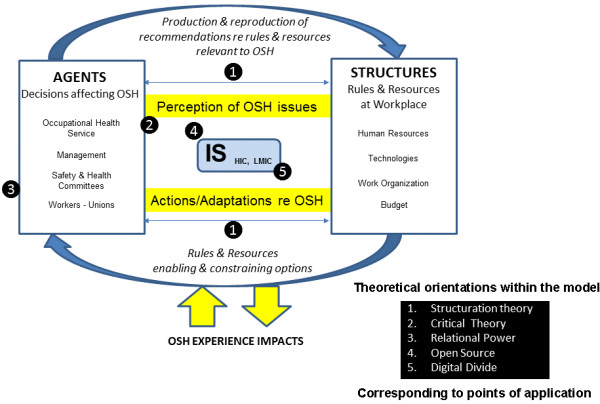
Adapted structuration model guiding analysis of Information System (IS) use for Occupational Safety and Health (OSH).

Finally, while pronounced “management–employee” power differences are manifest in all workplace settings, the right to be informed and involved in addressing relevant health determinants, especially through bipartite management-worker occupational health and safety (OHS) committees, is guaranteed by statute in a large number of jurisdictions worldwide [27]. However, although such “agents” (as we refer to this unit in Figure 1 below) exist in Canada [28], the United States [29], the United Kingdom [30], and Australia [31] as well as in European [32] and Asian countries [33] in addition to LMICs (including South Africa [34]), it must be acknowledged that they are not universal, and tend to be weaker in non-union environments and particular subsectors such as homecare within the healthcare sector.

Figure 1 presents the basic relationships that we set out to explore in scrutinizing “IS implementation relevant” power relations in workplace settings. The theoretical orientation that we apply can be characterized as an “adapted structurationist” model, allowing for greater exploration of power asymmetries within the workplace as well as at a global level. Adopting this approach, our article examines how contextual factors, and specifically power relations, can play a fundamental role in affecting IS implementation. In this sense, while applying a structurationist orientation to the “dualistic” influence of structure and social practices, we investigate the particular sensitivities to power relations inherent in this dialectic, as they relate to the various challenges highlighted above and integrated within Figure 1.

### The health sector workplace

The health sector constitutes one of the largest sources of employment worldwide [35], with acute shortages of health workers a serious concern, especially in LMIC settings [36]^a^. Healthcare systems worldwide are not only plagued by difficulties in recruitment and retention, but also biological, chemical and physical occupational health hazards as well as ergonomic hazards and psychosocial factors that lead to a high risk of injuries, illness and stress [35]. Nevertheless, despite the increasing use of online information systems in occupational health generally [37], and the existence of OHS committees that crave information for decision-making [38], and despite the fact that the health sector has a culture in which health-related surveillance is recognized as important, the use of IS has been rarely applied to improve the sector’s own work environments. The impact of globalization can, at least partly, explain this inattention to the well-being of the healthcare workforce – with increased casualization of work, subcontracting services and weakening of healthcare unions [39]. These factors also create a disincentive to reporting of hazards, let alone addressing these. As such, underreporting has been identified as a serious problem hindering accurate surveillance and appropriate action [40,41].

Accordingly, examining situations in which IS innovations have indeed been implemented in the healthcare workplace provides an excellent opportunity to analyze contextual influences in circumstances where other factors (knowledge, technical capacity, focus, recognition of rights) could support successful implementation. Acknowledging the power asymmetries in this setting, we particularly examine how an IS could serve (or be perceived) as a tool towards promoting the health of the healthcare workforce, or a weapon in service of other objectives. The objective of this study was to draw lessons from our own experience implementing information systems to address the health of healthcare workers in various contexts over two decades.

## Methods

We analyzed our team’s experiences over two decades in chronological order, first: i) in Canada – the provinces of a. Manitoba and b. British Columbia; and then ii) in South Africa – a. the province of Free State and b. the National Health Laboratory System (NHLS). We also reflect upon recent further international collaboration that has been developing to address the challenges identified. Given the prominence of the Canadian and South African experience in informing international efforts currently underway [42,43], and the leadership role these activities are playing within the World Health Organization (WHO) and International Commission on Occupational Health (ICOH)’s Scientific Committee on Occupational Health for Health Care Workers [44], an in-depth analysis of these experiences is especially warranted.

For this analysis, the context-mechanism-outcome method [45] was applied, in which we describe the socio-political context of each of the four initiatives, delineating the mechanisms employed to achieve success, and analyzing the outcome. This is the general approach used in “realist reviews” [45] - a strategy for synthesizing research that has an explanatory rather than judgmental focus. In realist evaluation, to infer a causal outcome (O) between two events, one needs to understand the underlying mechanism (M) connecting them and the context (C) in which the relationship occurs, with the basic evaluative question of ‘*what works*?’ replaced by ‘*what is it about this program that works for whom in what circumstances*?’ [46]. Mechanisms, moreover, are sensitive to variations in context, as well as to the operation of other mechanisms in a particular context [47]. We explicitly apply a structurationist theory approach sensitive to power relations in each of these contexts.

Several sources of information were used for constructing the C-M-O (Context – Mechanism – Outcome) analysis, as is summarized by Table 1. This encompassed: a) articles published about the context, the implementation of the system or from using data generated by these initiatives; as well as from b) surveys of users, c) key informant interviews and d) our own observations as researchers either directly involved in designing and implementing the systems in question (author AY), using the data produced (authors JS, AY, KL and LO), or studying the implementation of the systems in question (authors CD, AW and JS), as outlined further in Table 1.

**Table 1:** Summary of IS implementation review data sources

	**1.**	**2.**	**3.**	**4.**
**Setting**	Winnipeg, Manitoba, Canada	British Columbia, Canada	Free State, South Africa	Nationally, South Africa
	IS established at a large health facility with bipartite OHS committee	IS established at provincial level governed by bipartite board	IS built & piloted at hospitals in province with bipartite oversight	IS applied in a multi-site (349 labs) national institution
**Observation Period**	1986 - 1999	2000 - 2011	2007 - 2012	2010 - 2012
Data Sources				
**a. Articles reviewed**^**a**^	18 peer review articles	23 peer review articles	4 peer review articles	1 peer review abstract
**b. User surveys**^**b**^	Captured by participant observation	Manager & front line worker surveys	Pilot study interviews; survey conducted	Preliminary survey
**c. Key informant interviews**^**c**^	Captured by participant observation	Interviews of managers from 2 health regions & the provincial OHS regulator	Interviews of 2 OHS champions & other managers	Interviews of IS & OHS managers
**d. Participant-observer analyses**^**d**^	Researcher leading design & use; regulator perspective	Researcher leading design & use; research manager	Researcher co-leading design & use; research manager & coordinator; evaluator	Researcher co-leading design & use; evaluator

Notes: Details associated with notes a, b, c and d are provided in the Appendix.

Rather than detailing specific experiences, we focus on distinct characteristics associated with variations in the different applications that our team has pursued over the years, in order to especially assess the influence of contextual factors, and specifically the power relations within these. As was the approach of Porter in studying power relations between nurses and physicians in healthcare [48], the method of analysis we used could be called analytical induction, whereby tentative hypotheses about the power relations in the work environment were constantly refined, altered or abandoned in the light of the data collected. As noted by others [49,50], the use of participant -observation allowing direct observation of interaction has advantages over second-hand accounts in that what people *say* in the social setting of an interview may be considerably different from how they actually *behave* - and the information elucidated from mundane events which the social actors may not recount to an interviewer [50] can provide valuable insights.

Ethics approval for conducting IS-related research associated with the cases conducted in British Columbia and South Africa was granted by the University of British Columbia, with approval in Free State also issued by the University of Free State. Research in Manitoba had been carried out with ethics approvals from the University of Manitoba^b^. Combining formal studies (see footnotes and references for itemization of various surveys conducted, key informant interviews, and publications consulted) with two decades of participant-observations from members of the research team allows for strong triangulation.

## Results

The results from the C-M-O analysis of the four case studies are presented below and summarized in a tabular format, with a further summary provided according to the micro, meso and macro-level context of each case:

### The Experience in Canada

#### 1a. Manitoba (1990–1999)

A database on occupational health for healthcare was constructed in the province of Manitoba, during the 1990s – the first of its kind in Canada [51]. As summarized in Table 2, rudimentary technology was used in a partnership between our then University of Manitoba-based team and the Winnipeg Health Sciences Centre (HSC), at that time Canada’s largest integrated healthcare complex, to collect data related to causes and contributors to work-related disease and injury in healthcare workers [52], as well as immunization rates [53], and the effectiveness of interventions [54]. Considerable success was achieved [55]. The Winnipeg HSC was well resourced with a strong occupational health team and a functional joint worker-management OHS committee. The occupational health practitioners served as resource-experts *ex officio* to the bipartite committee, and as such, were trusted by both workplace parties.

**Table 2:** Context-mechanism-outcome (C-M-O) summary of workplace health IS use - Manitoba, Canada

Context	Surveillance system - created to assist occupational health (OH) department’s health professionals in a large (7,000+ workers) well-resourced teaching hospital with their primary and secondary prevention activities as well as for implementation research (database not containing fields necessary for claims cost containment).
	Bipartite (union-employer) health and safety (H&S) committee supportive; labour relations amicable.
Mechanism	*Governance:* Developed under auspices of a university-hospitalpartnership (which informed a bipartite H&S committee), with an affiliation agreement in place for collaborative research and service.
	*Technology:* Easily accessible; existing standard software (Excel, Access databases).
Outcome	Decrease in injury rates and time loss due to injuries demonstrated; information also used to improve vaccination programs, and foster good research.
Conclusion	Useful and sustainable, albeit limited to one workplace as system not web-based, and screens not optimally user-friendly, so required commitment to data collection and data entry.
	“Tool”, but not a highly efficient one. The power dynamics were such that the risk that the IS would be used as a “weapon” was minimal.

Thus, at the micro level (personal relationships on a day-to-day basis), the fact that the database was designed and governed within a clear university-hospital partnership headed by a single individual with dual responsibility (co-author AY) made the use of the system both for research and operations quite smooth; at the meso level (stakeholder relations within the hospital), interactions were cooperative, with power imbalances between workers and employers adequately mitigated by the OHS Committee (the “agent” in this regard); and at the macro level (general socioeconomic and political conditions), the context was very supportive for IS introduction with the provincial social democratic government quite enabling. Table 2 summarizes the key context-mechanism-outcome relationships that the authorship team synthesized from the various articles written, and participant-observer experiences. There was no overt power struggle that interfered with the development, implementation or use of the IS, albeit the tool was limited by lack of technological sophistication.

#### 1b. British Columbia (2000–2010)

In 1999, the Occupational Health and Safety Agency for Healthcare (OHSAH) was created in another Canadian province, British Columbia (BC), as a bipartite union-employer collaborative agency, and programs were developed from this perspective [56]. In designing a specific database to monitor and evaluate overhead lifts to prevent musculoskeletal injuries to healthcare workers [55], it became clear that prevention requires more than *tracking* injuries – it calls for occupational health practitioners and active involvement by frontline workers. This lesson, regarding the importance of carefully considering the perspective amongst the different parties also came across clearly in the evaluation of the Prevention and Early Active Return-to-Work Safety program [57,58] in which an analysis late in the program showed that the program theory differed considerably amongst the worker, the employer and the practitioner stakeholders [59]. Building on the experience in Manitoba and advancements in the internet, a web-based system was developed, called Workplace Health Information Tracking and Evaluation (WHITE) [60,61]. The context, however, was quite different. Although BC is a wealthier province than Manitoba, according to a formal needs assessment of occupational health resources conducted for a provincial agency, occupational health staffing was “deplorably” lower than international norms [62], labour relations were more volatile, the power imbalances more pronounced and the governance of WHITE was unclear.

Interviews with stakeholders conducted more than five years after implementation of WHITE demonstrated strong commitment to the integration of occupational health data collection across health regions in BC to comply with reporting regulations, and to improve compensation claim and disability management. However, there was an apparent lack of collaboration between system users, management, and system developers. Moreover, it seemed to us that the employers were more interested in the claims management aspect to reduce costs than using the data to promote bipartite collaboration supporting workplace hazard reduction [61], let alone empowering the workforce. In 2010, OHSAH was discontinued as a bipartite agency, and WHITE, with no clear rights entrenched for researchers or frontline workers to access the data, was transferred to the IT department of the provincial health authority.

Thus, at the micro-level, the fact that the database was designed and governed at the provincial level, but the data were meant to be gathered and used at the (understaffed) health facility level was problematic; the meso level was complicated by unclear governance regarding rights to access and use of data; and at the macro level, the conservative political environment and weak unions made it difficult to use the system for empowering the workforce. Table 3 summarizes the key context-mechanism-outcome synthesized for this case. The power asymmetries in this case did interfere with use of the IS; albeit the IS served as a useful tool to control claims costs, its usefulness to OHS committees, as noted previously [61], was more problematic.

**Table 3:** C-M-O summary - British Columbia, Canada

Context	Workplace health information tracking and evaluation system (WHITE) developed within a bipartite healthcare agency for all health sector sites in a wealthy province, but site-specific OH departments poorly resourced.
	Labour relations volatile and unions relatively weak in this period.
	With time, collaboration between system users (practitioners), management and system developers became weaker, with no systematic use by H&S committees.
Mechanism	*Governance***:** Developed by a university researcher working with bipartite provincial agency, but no affiliation agreement between the agency and university partner; later the IS was transferred from the bipartite agency to employer-control, with no rights to access for researchers.
	*Technology:* Customized web-based software developed, using proprietary database technology.
Outcome	Decrease in injury rates and time loss due to injuries demonstrated; information has not been able to be sustainably used to improve workforce health, or initiatives such as vaccination programs, nor foster ongoing research.
Conclusion	Useful to employers for ongoing claims management,
	Limited use of data either to promote bipartite collaboration for reducing workplace hazards or to support programs that require OH staff in place, or for ongoing high quality research.
	Web-based system made it useful across entire province, including multiple workplaces, but expensive to maintain.
	“Tool” for employers and regulators;
	“Weapon” to busy OH practitioners who are stretched to their limit with no time for data entry, and possibly to workers concerned that the greater ‘efficiency’ in absenteeism control and time-loss reduction could hurt vulnerable workers in a climate of weaker job security.

Comparing the asymmetries of power in labour relations between the BC and Manitoba experience points to the importance of ensuring that the purpose of the data system be carefully monitored to ensure that its use is not diverted to alternative objectives (e.g. from initially empowering workforces to take action to improve their health and safety, toward facilitating employers’ ability to reduce claim costs) [61].

### Collaboration in South Africa

While concerns about the healthcare workforce are global, the greatest need for health workers and their protection is in Africa, where the WHO estimates that the workforce will need to increase by 140% before a critical shortage is overcome [35]. Furthermore, health workers are at higher risk than the general population for tuberculosis and other infectious diseases [63], where such illness is creating additional staff shortages [64], contributing to increased stress, burn-out and related health risks [65]. The increased workload is in part attributable to the high burden of HIV/AIDS in the country as well as the risk for infection to health workers (a national HIV prevalence of 15. 7% among South Africa’s nurses [66]). Meanwhile HICs have actively encouraged emigration of health workers [67], only aggravating a difficult situation. In addition to the fact that South Africa currently faces a major crisis in terms of human resources for health, there are skewed distributions between the public and private sectors [68].

Building upon the experience in the two contexts in Canada, and from interactions through the WHO collaborating centre network [69] where the collaborating partners first met, the WHITE system was re-designed and re-named the Occupational Health And Safety Information System (OHASIS). Unlike its Canadian predecessor, OHASIS explicitly provided modules to help build capacity of joint worker-employer OHS committees and to improve working conditions; and unlike its Canadian predecessor OHASIS was not built with a claims management module. The explicit purpose of OHASIS is to provide workplace and workforce surveillance data for operational decision-making in improving working conditions and over-all worker health, in line with government policy in this regard, while at the same time serving as a treasure of data for local and international research on determinants of injuries and illness in health workers.

#### 1a. Free State (2006–2011)

The pilot launch of the South African OHASIS collaboration occurred in one large regional Hospital in Bloemfontein, Free State, and included input from national and provincial decision-makers as well as occupational health practitioners and health worker trade unions [70]. This site was selected for several reasons: In 2006, the Free State Department of Health had established a task team to coordinate and evaluate occupational health activities in the province, thereby confirming commitment for improving this area; the province had experience with information systems in broader population health applications [71] and there was strong management and union support at the local level, as well as interest by the local university. Most importantly, though, the collaboration between Canadian and South African experts began precisely with a focus on how to build capacity of health workers to address their working conditions [70] with the data system to target this objective.

A feasibility study^c^ conducted prior to launching a formal pilot study evaluation [70] found that several improvements were needed in the system– to make it more user-friendly and robust for data analysis. These changes were then made, but as time had passed, another baseline assessment had to be conducted to serve as a comparison when implementing the newly redesigned system. This second survey, conducted in 2010, assessed frontline workers’ knowledge, practices, and attitudes around infection control and working conditions, and was completed by 110 participants at the targeted Hospital. In 2012, the survey was re-designed and re-administered to health workers at this hospital and the sampling frame was expanded to include respondents from two other large hospitals located in the Free State province. The full results with South African collaborators will be reported elsewhere, but for purposes of this analysis it is noteworthy that respondents of the second survey were divided in their perception of how easy it was to obtain information about potential workplace risks – 37% found it easy, 39% stated it was not easy, and 22% answered that they did not know. With regard to the dynamics of power within the hospital, contrary to the international team’s impressions that strong support from management was present (possibly in comparison to the situation in Canada), 41% of 109 staff respondents reported that they did not feel adequately supported by their managers in matters pertaining to health and safety; 12% reported that the management/hospital could never be trusted, while 31% said that they could be trusted a quarter to half of the time, with only 38% saying that the management and hospital could always be trusted. This is especially relevant when it comes to workforce health information; although OHASIS has solid security features to guard confidentiality of personal, it is understandable that lack of trust would undermine support for the system. Furthermore, many key informant interviews uncovered themes around the imbalance of power between management and workers. In discussions regarding the divide between workers and managers, some workers indicated that while there is organizational rhetoric around keeping employees safe, in actuality, prevention measures were lacking. Additionally, by the time the re-designed system was ready for usage, the political context was no longer optimal for both implementation.

In August 2010, a bitter public sector strike occurred in South Africa that dramatically affected the labour situation in the hospitals. Amidst this heightened union-management acrimony, one of the unions decided that until a properly elected OHS committee was established and adequately trained from their perspective, the project should not go ahead. Albeit reluctantly, the formal launch of the IS planned for Free State had to be delayed. In September 2011, the issues were resolved, allowing implementation of the new version of OHASIS to go forward. This proceeded well, with logistical deployment issues (e.g. ensuring adequate connectivity among users) then addressed. However, in implementing at a second hospital site, new challenges emerged, attributable at least in part, to the legacy of racial tensions, as well as possible concerns about sharing data from the hospital with the provincial level, where OHASIS was hosted.

Thus, at the micro-level, the on-site OH practitioners and the researchers all wanted the IS, there were excellent relations established and the system was designed for workforce empowerment. At the meso level, however, considerable barriers occurred due to labour concerns about governance, which also had to be resolved, and other issues related to power imbalances had to be addressed through additional clauses to the initial memorandum of agreement. At the macro level, the dependence on the Northern partner for sustainable IS technical support emerged as a concern, with the original vision of an ongoing relationship with an operating IS system in an HIC setting (i.e. the WHITE system in BC) no longer viable. The solution for this came from involving a WHO partner at the South African national level, as is discussed below. Table 4 summarizes the key context-mechanism-outcome relationships the authorship team synthesized from the articles written, interviews, the survey noted above and the extensive participant-observer experiences.

**Table 4:** C-M-O summary - Free State, South Africa

Context	IS (OHASIS) developed to increase capacity for improving working conditions in South Africa healthcare, launched as pilot in resource poor setting but with more human resources devoted to OH than in BC (comparable to the Manitoba setting).
	Initially strong bipartite H&S committee support, but political changes, heightened racial tensions, increased union militancy and complex governance concerns created challenges
Mechanism	*Governance*: Partnership between Canadian and South African university-based researchers, with provincial health department.
	*Technology*: Similar to the BC system, but with module development emphasizing prevention and capacity-building OH activities.
Outcome	While feasibility study was positive, and OH professionals keen to use system, implementation of revised system was delayed due to political power struggles (union discontent with how joint health and safety committees were established).
Conclusion	System designed for prevention and empowerment of the workforce; delay in implementation because of expressed union concerns.
	“Tool” to OH practitioners, however efficiencies not realized, as new system with improved reporting functions was never implemented in this time frame.
	“Weapon” use by union militants to leverage achieving other demands.
	“White elephant” to the researchers and decision-makers who invested in the system, and so far do not have a usable system implemented.

#### 2b. The National Health Laboratory System (NHLS) (2009–2012)

With unions at the national level keen to see OHASIS adopted, the NHLS did not experience the same political constraints as were experienced in Free State. Accordingly, implementation of OHASIS across all 350 laboratories across South Africa was initiated in mid-2011, operated by the NHLS, with NIOH serving as technical advisors. NIOH also agreed to provide technical support to Free State, which helped resolve both technical and political concerns that arose with the system maintained from Canada, alluded to above. Details of this experience will be presented separately regarding both the experience within NHLS [72] as well as the implications for the Free State implementation. Table 5 summarizes the C-M-O analysis conducted from the experience to date.

**Table 5:** C-M-O summary - National Health Laboratory System, South Africa

Context	Same tool (OHASIS) adapted to increase capacity in healthcare laboratories across South Africa to improve working conditions.
	Personnel accustomed to computerized data collection, resources devoted to OH staffing, and union support present at the national level.
Mechanism	*Governance*: Partnership between Canadian and South African researchers affirmed in writing.
	*Technology*: Similar to above two, but with more user-friendly features.
Outcome	Being implemented in 350 laboratories across South Africa, but sustainability of system still questionable, as IT department still depends on northern partner, but transition plan in place.
Conclusion	Even when a system is successfully launched, and labour relations are supportive, IT capacity-building is essential from the outset to ensure sustainability.
	“Tool” to all, but risks becoming a “White elephant” if the IT capacity can not be quickly built to take over full maintenance and further development.

The context of implementing the workforce health information system was thus very different in South Africa compared to Canada. With militant trade unions in healthcare, attention to union demands had to be a pre-requisite to successful implementation. Moreover, despite the fact that the Southern practitioners had initiated collaboration with the Northern researchers, the North–South collaboration was still somewhat perceived as threatening in the less-developed less-well-resourced setting, but was openly welcomed in the better-resourced WHO-affiliated centre, NIOH. The mechanism for successful implementation was indeed the WHO collaborating centre network, which allowed the building of trust – key for success, and facilitating the empowering of a locally-based WHO collaborating centre looking after the IS needs in this field within not only South Africa but the entire African region^d^.

Table 6 summarizes our analysis of how factors related to context and mechanisms affected the contribution of IS to achieving outcomes in the four cases presented.

**Table 6:** Summary of contexts and mechanisms needed for successful outcome at different levels

	**Man**	**BC**	**FS**	**NHLS**
**Micro**				
Commitment to health and safety including adequate staffing to plan, implement and evaluate interventions	**+**	**-**	**+**	**+**
**Meso**				
Clear governance (access, use of data)	**+**	**-**	**+**	**+**
Good labour relations so neither side is motivated to use the IS as a “weapon” rather than “tool”	**+**	**-**	**-**	**+**
**Macro**				
Enabling political environment	**+**	**-**	**+**	**+**
Sustainable local IT capacity (so system does not become white elephant)	**-**	**+**	-*	**+**

*based on the analysis conducted for this study, efforts are now underway to implement mechanisms for sustaining needed capacity, in the form of international networks and regional WHO Collaborating Centres taking on leadership roles in their jurisdictions.

The specific characteristics, outlined in the form of a checklist in Table 7, suggest criteria that should be considered when implementing technical solutions to address social health determinants generally.

**Table 7:** Power-relations checklist for implementing occupational health information systems (IS)

MICRO**:** WITHIN THE WORKPLACE OHS DEPARTMENT **–**	
***WHO MIGHT BENEFIT OR FEEL THREATENED BY THE IS?***	
□	Will only specific occupational health practitioners enter data and access system? Or all? If only some, is there good consensus on this amongst the OHS personnel?
□	What will be the impact on staff workload? Is staffing adequate?
□	Are personnel adequately trained to capture data correctly?
□	Will health and safety representatives be able to access any aspects of the system?
□	If so, are they trained adequately?
□	Are all appropriate personnel trained to interpret and act on the data?
□	Who will receive aggregated reports?
□	How often will aggregated reports be generated? Who will write commentaries?
□	Is the local technology adequate – (i. e. computers, bandwidth, etc. )?
□	Have policies and procedure been written to guide system use, confidentiality of data, and access to reports?
□	Is there a communications plan established between system implementers and the workplace staff who will use the system?
MESO: WITHIN THE ORGANIZATION	
***WHO MIGHT BENEFIT OR FEEL THREATENED BY TH IS?***	
□	Have the unions or worker representatives been adequately consulted about the introduction of such a system?
□	Were frontline managers adequately consulted about the introduction of such a system?
□	Was the information technology department of the workplace adequately involved?
□	Did all the appropriate workplace parties have input to the design, policies and procedures regarding use of the system?
□	Are all the workplace parties throughout the organization aware of how they might benefit from the system?
□	Are there clear channels of communication between units within the organization to ensure equity and foster shared involvement/ownership?
MACRO: BEYOND THE ORGANIZATION	
***WHO MIGHT BENEFIT OR FEEL THREATENED BY THE IS?***	
□	Who designed the system? If the design occurred out of the jurisdiction where the IS is being implemented, were local stakeholders adequately involved in adaptations?
□	Is the governance of system use and financing clear?
□	Who governs the maintenance, upgrade, or system design modifications?
□	How is the maintenance and upgrade of the IS being financed? Are the financial benefits fair?
□	Are the terms and conditions sustainable even if the current decision-makers and/or technical personnel all change?
□	What aspects of the local, regional, national or international political climate may impact the system? If a less worker-friendly government come in, will this impact system use?
□	If financial issues and governance involve multi-scalar (i. e. hospital- province/state –national-international) cooperation, what other political issues may arise and how can these be managed?

## Discussion

Building on the observation that IS can change the organizational landscape of power and status [73], Tjora & Scambler [9] suggest that a factor explaining why IS innovations have been disappointing lies in IS having ignored the importance of “role” within an organization. Our analysis of factors affecting IS implementation, consistent with that of Sein and colleagues [74] also shows that the distinct interests and roles of different sets of actors are critical in shaping IS introduction and use – and affect the dialectic that our adapted structurationist framing of IS implementation seeks to explain.

Amid mounting interest not only in the health of health workers but more broadly in the significance of social determinants of health, systematic consideration of how data systems affect power relations is warranted. Innovations such as IS applications for conducting inspection activities [75] providing more comprehensive and timely information to health professionals and managers [76] or establishing more comprehensive general surveillance of affected communities [77] suggest ways that experts can extend their consideration of health-relevant information. Nonetheless, direct engagement of the people whose health is being monitored has remained neglected – leaving them *de facto* as little more than *objects* for observation.

There is worldwide consensus on the need to improve monitoring and evaluation of the health and safety of health workers, especially given the unrelenting challenges of HIV and tuberculosis [78]. As for health information systems generally, leadership and drive has primarily come from professional, expert or managerial champions, often acting in concert with proprietary commercial developers and providers [79]. A review of commercial software systems implemented to address the health sector workforce, in fact observed that these have tended to focus on providing information for financial and administrative managers, with limited usefulness to surveillance of workplace risks [80]. Our analysis of the power relations helps explain the skewed development of IS in this setting, and the importance of new initiatives underway internationally.

The incentive structures reinforced by how intellectual property and issues related to licensing have defined the context for developing IS solutions for OHS issues most highly valued by financial and administrative managers must, however, also explicitly be taken into account. To ensure that the potential benefits of IS solutions are not limited or skewed by proprietary ownership, IS innovations have also triggered development of alternative licensing and knowledge-sharing orientations. As noted [69], in the spirit of seeking such solutions with international partners and mitigating potential power imbalances, our team has explicitly pursued the model of Creative Commons licensing described above, and is actively pursuing further software refinements with the South African and other International partners.

Exploring further implementation opportunities, in 2009, our University of British Columbia-based team joined an international collaboration linking the Pan American Health Organization (PAHO), Health Canada, the Ecuadorian Ministry of Public Health, and Vancouver Coastal Health Authority (VCH) to strengthen Ecuador’s capacity to promote healthier and safer healthcare working conditions [81]. The project fit under the framework of the PAHO Regional Plan on Workers’ Health [82], as well as the WHO Global Plan of Action on Workers’ Health [83], which explicitly urges member countries to implement occupational health and safety policies and programs in the healthcare sector. The interdisciplinary team selected three public Ecuadorian hospitals and conducted a baseline assessment in thirteen medical units from across these three facilities [84]. Building on a well-established collaboration between the Canadians and Ecuadorians [85] efforts were pursued with government and hospital personnel and academic partners to adapt the IS for health workers in Ecuador, linking this with support provided by the Andean Region Health Observatory maintained by the Andina Simon Bolivar University [86]. Other countries have expressed interest in OHASIS, and efforts are now underway to make this available through our Ecuadorian-based partner, using a Creative Commons license encompassing the three principles noted above.

Meanwhile, the US National Institute of Occupational Safety and Health (NIOSH) very recently created a portal to collect information in a central repository – not only from within the US, but internationally; OHASIS was then re-designed to be able to not only export into the NIOSH repository, but the OHASIS team has created a user-friendly interface to facilitate the adaptation of other systems to contribute to this data pool to facilitate international collaboration. It is also noteworthy that OHASIS contains EpiNet^tm^ within it [87] – modules developed to specifically capture information on needle stick injuries and other blood and body fluid exposures – used freely in dozens of countries worldwide, also operating within the WHO network.

The findings from our realist analysis of two decades of experience show the extreme relevance of considering power relations in the micro, meso and macro contexts of healthcare *workplace* setting, where power dynamics are of considerable importance, and access to information on workplace hazards, occupational injuries and illnesses, as well as information about the risk factors, and health of health workers can be quite contentious [61]. The lessons learned in this regard are summarized in Table 7 in the form of a checklist for consideration in pursuing implementation. Although experts have previously noted that ignoring political realities and organizational social dynamics can lead to IS failure [88], this is the first study that examined these factors in determining IS use to promote workforce health.

Efforts to make information about occupational health risks and their controls available online are increasingly being embraced [37], in keeping with evidence that empowerment of the workforce (and OHS committees) is of paramount importance in improving workplace conditions and worker well-being [89], and requires proper information and training [90]. Furthermore, research shows an important link between trust, management supportiveness and safety culture [91]. Accordingly, we began our work in Free State with workplace audits, and assessments of worker knowledge, attitudes and self-reported practice, and then incorporated a module on workplace assessment into the IS itself, thereby addressing a specific surveillance need noted by Gaydos and colleagues [75]. Our analysis, nevertheless, suggests that even attempts to address this weakness can still be insufficient to overcome the impact of the power asymmetries in workplace settings.

## Conclusion

As we argued more than twenty years ago [92], a surveillance system is more than a data collection system – it requires the ability to interpret and act on the information as part of an inherently iterative monitoring function. This capacity-strengthening focus is what makes an IS in this setting such a potentially powerful tool.

Nevertheless, meticulous attention is needed to address power asymmetries at different levels: micro (e.g. worker – management relations within the workplace, including addressing the disincentives to reporting hazards and incidents, empowerment of OHS committees and adequate staffing); meso (including governance issues, role of university partners and North–south team dynamics in the absence of sufficient capacity-building); and macro (including political climate, proprietary licensing and global patent protections). North–South-South partnerships (i.e. where an HIC partner facilitates direct collaborations between LMIC partners) should be encouraged, and are showing promise. In this regard, making IS solutions that are developed in HIC settings available for application and further collaborative development in LMIC contexts, as was initially planned, can substantially reduce cost burdens and broaden a “creative commons” network and community of practice to enhance sustainability of IS uses in LMIC settings.

If the power asymmetries in the mechanisms established for implementation are not properly addressed, however, the system, while meant as a *tool*, can be seen as a *weapon* (an instrument of control exerting power of one set of agents over another). Such perception can come from many sources: by busy healthcare professionals who feel pressured into collecting and entering data into a system that provides them minimal benefit; by a workforce that is skeptical that confidentiality will be maintained and/or concerned that the information will be used to empower managers as absenteeism police; or indeed, by union activists, who may wish to use this tool for purposes which the system was not meant to address. Furthermore, of particular importance in these resource-constrained times, the system, with its technologically sophisticated requirements in settings not habituated to this, can easily become a white elephant. A structurationist analysis, reinforced by explicit consideration of these power dynamics, applied at micro, meso and macro levels, is therefore especially useful when embarking on IS implementation.

## Endnotes

^a^We adopt the World Health Organization definition: “*Health workers are all people whose main activities are aimed at enhancing health. They include the people who provide health services -- such as doctors, nurses, pharmacists, laboratory technicians -- and management and support workers such as financial officers, cooks, drivers and cleaners. " WHO Fact Sheet #302*. April 2006 (http://whqlibdoc. who. int/fact_sheet/2006/FS_302. pdf). Nonetheless, the shortage of health workers globally does not apply to all categories of health workers.

^b^ This includes UBC ethics certificate # H10-00360-A003 and H05-80551. Other certificate numbers, including those for studies more than 5 years old, are available on request.

^c^ In September 2007, the NIOH and the South African Health Department organized a seminar attended by national and provincial occupational health coordinators and Canadian team members. Almost 100 participants attended this initial workshop, including health and safety representatives, occupational health and infection control professionals, union activists, clinicians, managers and academic collaborators. Here, a standard core curriculum, core competencies, and a methodology for rolling out a training program for health and safety representatives in South Africa was discussed. The research and training materials were developed based on expertise gained from work conducted by team members in Canada, South Africa and Ecuador and included a training guide, problem-based learning case scenarios and a workplace audit tool. Topics covered in the training included: roles and responsibilities of occupational health professionals and health and safety committees, basic concepts in occupational health (i.e. the hierarchy of control measures), incident reporting and investigation, workplace assessment and workforce health. These materials were subsequently used each year to train new health and safety committee members at Pelonomi Hospital. All workshops were formally evaluated and materials and format were amended as necessary.

^d^ A memorandum of agreement was signed November 30, 2011 between the University of British Columbia and the South African NHLS to allow free transfer of the system, including source code, with the explicit understanding that the South African partner would provide support for OHASIS implementation and use across the African region.

## Appendix Data Sources Details (notes for Table 1)

^a^ These studies, ranging from analyses of the epidemiology of injuries to various occupational groups of health workers (nurses, food handlers, laundry workers, etc.) to determinants of various types of injuries (needlesticks, musculoskeletal injuries, violence, etc.) to effectiveness of various programs (return-to-work programs, musculoskeletal injury prevention initiatives, vaccine programs, etc.) were reviewed by at least two members of the research team. 

In addition, articles that describe the context were reviewed, including those that detail the history and challenges in the Canadian Institutes for Health Research (CIHR) Community Alliances for Health Research (CAHR) program that funded much of the collaborative research with the institutions involved e. g. [56,93] were also reviewed.

^b^ These included surveys prior to implementation (e. g. of managers at Fraser Health in BC [94], and of front-line health worker e. g. [95]). 

In addition to the surveys referenced in the text related to the NHLS implementation, a separate publication will be submitted next year detailing the results of the survey conducted at three hospitals in Free State.

^c^ Key informants specifically interviewed in formal interviews included senior managers as well as personnel in charge of prevention in BC; in South Africa, occupational health practitioners were formally interviewed as well. 

Specifically, co-author CD conducted one-hour semi-structured key informant interviews in August 2010 with two occupational health champions working in Bloemfontein, South Africa. 

Due to the nature of the information solicited, identifying details about the titles of the informants cannot be given, however, each person worked at two key hospitals in the area. 

One person worked in OSH and was assisting in the implementation of OHASIS at their hospital, while the other provided care to health workers who experienced OHS events in the hospital. 

Both were able to speak to the complex power dynamics that both facilitated and impeded the flow of OHS knowledge between health workers, their unions, management and the executive leaders of their hospital. While our team led research and implementation planning meetings and activities, two project team members observed interactions between staff of different levels over a full month in August 2010. 

This qualitative data was also thematically analyzed. Co-author AW conducted one-hour semi-structured key informant interviews with various professionals involved in the implementation and use of the IS in BC in April and May 2010: interviewees were from two different health regions and Work Safe BC. 

(To protect the identities of the interviewees, titles and positions in their organizations are not provided here.) 

AW also conducted a thematic analysis of the qualitative data. Additionally, AW spent a few months in Free State in 2010 assisting in the design of the initial survey and data collection and analysis.

^d^ Co-author AY was the Founding Director of the occupational health department in which the Manitoba system was developed and implemented (1986–1999), as well as research director for studies conducted during this period using the IS. 

AY was also the Founding Executive Director of OHSAH as well as the Principal Investigator (PI) of the research program in BC (1999–2007) in which the BC IS was developed and implemented. 

She also is PI of the research program in which the South Africa system was developed and is being implemented (2007-ongoing). 

Her experience and reflections constitute an important part of the fieldwork for this study. 

Co-author JS is a program evaluation and policy researcher with over 30 years of experience in various university and government regulatory and policy (including Manitoba and BC) settings in examining effectiveness of occupational and environmental health systems and interventions. 

He is PI of a CIHR funded comprehensive study of the implementation of the information system in South Africa. 

Co-author KL served as Research Manager of the CAHR in BC from 2003–2007, and is now the Research Manager for the research program in South Africa. 

Her experience in implementing the IS and using data collected is thus also an important source of insight for this article. 

Co-author LO conducted 10 fieldtrips to South Africa in which the design and implementation of the IS were key topics of observation. 

She has spent over 9 months total working with occupational health practitioners, clinical managers, provincial decision-makers and other stakeholders in this regard.

## Abbreviations

BC: British Columbia; C–M–O: Context – Method - Outcome; HICs: High Income Countries; HSC: Health Sciences Centre (Winnipeg Manitoba Canada); ICOH: International Commission on Occupational Health; IS: Information System; LMICs: Low and Middle Income Countries; NHLS: National Health Laboratory System (South Africa); NIOH: National Institute of Occupational Health (South Africa); OHASIS: Occupational Health And Safety Information System; OHSAH: Occupational Health and Safety Agency for Healthcare (BC Canada); OSS: Open Source Software; PAHO: Pan American Health Organization; WHITE: Workplace Health Information Tracking and Evaluation; WHO: World Health Organization.

## Competing interests

The authors declare that they have no financial competing interests. The information systems reported herein are non-commercial, and the source code for the Occupational Health And Safety Information System (OHASIS) is freely available.

## Authors' contributions

JS is the principal investigator of the “Tool, Weapon or White Elephant” study funded by the Canadian Institutes of Health Research, which helped form the conceptual basis of this article. He led in the conceptualization and writing of this manuscript, along with AY. He also personally did the final editing and preparation for submission. KL conducted a literature review to support the article, assisted in the initial drafting and contributed her observations from years of working with the information system in British Columbia. LO assisted with the drafting of the article based on her involvement with the system development and implementation in South Africa. AW assisted with the drafting of the article, as well as drafting and conducting the survey of system users in South Africa noted in this article, along with the collation and interpretation of the results; she also interviewed system users in British Columbia and incorporated these observations into the article. CD also assisted with the drafting of the article, as well as drafting and conducting the survey of system users in South Africa. She also conducted interviews of system users in South Africa and contributed these observations to the article. AY, the senior author on this manuscript, played the lead role in developing, adapting and implementing the systems described in all five settings, as well as conducting research related to system use in all five settings. She wrote the first draft of this article, and worked closely with Dr. Spiegel on the final draft. All authors read and approved the final manuscript.

## Pre-publication history

The pre-publication history for this paper can be accessed here:

http://www.biomedcentral.com/1472-6947/12/84/prepub
